# Annotations

**Published:** 1890-03-29

**Authors:** 


### Annotations.

**M. Pasteur at Play.**

The unscientific world that lives in outer darkness must often feel a keen curiosity as to how the tremendous personages who dwell in the giants' castles and on the loftiest mountain peaks of unattainable science amuse themselves. One of the most illustrious of those rare illuminati has recently shown the world a sample of the particular kind of amusement which he affects. As might have been expected, Pasteur at play is still Pasteur; and being Pasteur he is profoundly and exclusively "scientific." Dr. Camac Wilkinson, chairman of the Executive Committee of the Australian Rabbit Commission, gave an instructive account in last week's *British Medical Journal* of certain "Rabbit" experiments made by a body of French delegates, acting under M. Pasteur's orders at Sydney. The experiments, as most people are aware, were designed to show that rabbits could be infected with chicken cholera, and that the infected animals when turned loose among their healthy fellow rabbits would infect them in turn. By means of this infectious disease of chicken cholera, thus converted into rabbit cholera, it was hoped to exterminate the pest of rabbits in Australia. To the benighted and unscientific plain man it would seem as if nothing should be easier than to prove whether or not it was possible to exterminate the rabbits by such means. The first thing surely to do would be to catch your rabbit or rabbits; then to make sure that they were properly infected with the disease: then to turn them loose within a limited area, and await results. If all or any large number of the healthy rabbits of that area were to become infected and to die within a reasonable time, then it would be evident that there was some hope either of exterminating the rabbits completely by Pasteur's method, or at least of keeping their numbers within manageable bounds. The commission appointed by the Australian Government, of which Dr. Wilkinson was executive chairman, naturally expected, as all common sense persons would, that some such procedure would be followed. But Dr. Wilkinson reckoned without M. Pasteur. That gentleman evidently made up his mind to have a little fun at the expense of the Australians and their rodents. He sent out certain delegates, and like Philip of Spain with his Netherland subordinates, laid down for their guidance the most precise instructions, which were to be carried out in the most minute particulars. Those instructions were: (1) To feed rabbits with broth cultures of the microbe of chicken cholera. (2) To feed ordinary domestic animals in a similar way. (3) To make an experiment within an enclosed area of about 500 acres in a district thickly infested with rabbits. In this area the food of the rabbits was to be sprinkled from day to day with cultures of the microbes of chicken cholera. The Commission very naturally objected altogether to the feeding with microbe cultures. They did not doubt that if the whole of the Australian continent could be sown with such cultures a good many rabbits would die, any more than they doubted that a good thick sprinkling of phosphorus would be likely to cause a considerable mortality. Surely there was no need for delegates to go from France to show the Australian scientists that rabbits could be poisoned by being fed with cultures of the microbes of chicken cholera. One thing, and one only, was asked of Pasteur and his delegates—that was to make plain beyond doubt that rabbits infected with chicken cholera could promptly and extensively infect healthy rabbits so that there might be a reasonable hope of exterminating or thinning down those enemies of the colonists. That one thing Pasteur and his delegates failed to do. No only so, but by an authoritative cablegram the great scientist vetoed experiments suggested by the Commission for the purpose of proving or disproving the possibility of extermination by infection as opposed to direct poisoning. As all the world knows, a quarrel has ensued between the delegates and the Australian Commission. An impartial onlooker cannot help feeling that Pasteur, at any rate, whatever may have been the case with his delegates, has been indulging in a little scientific fun at the expense of the Australians and their rabbits.

**Specific Diseases: Infection.**

"The natural history of specific diseases" is not the easiest subject in the world to write about. In spite of its difficulty, Dr. Edward Willoughby has undertaken the task. The first condition of interesting and successful writing is knowledge. As Lord Beaconsfield once said, "Knowledge is the parent of eloquence." There is a good deal of knowledge available about specific diseases; unfortunately, there is also a good deal of ignorance, and that, too, is equally available. There are, in addition, a very considerable number of hypotheses in circulation. These, unhappily, are being frequently accepted as current scientific coin, exactly as if they were demonstrable knowledge. The man whose scientific pockets are stuffed with them, fancies himself scientifically rich, and expresses both disgust and anger when he finds the value of his hypothetical counters called in question. But he may be reminded that whilst almost any hypothesis is better than none, yet no unproved hypothesis can be permitted to take rank with demonstrated facts. There is an end of all science when hypotheses cease to be called in question. In considering such subjects as specific diseases, a critical attitude of mind is absolutely indispensable. What is infection, and how is it brought about? That is a plain question—a question to which any book on specific diseases ought to offer a plain answer, if such an answer be scientifically possible. But is it? The first chapter of Dr. Willoughby's book deals exclusively with infection. Dr. Willoughby attempts a precise explanation of the word and what it connotes. It is interesting to note the language which he uses. A specific disease, he contends, is always brought about by infection. It never arises *de novo*. As if afraid to commit himself fully and absolutely to the invariable "origin by infection" hypothesis, he says of specific diseases that "they never arise *de novo*, or, more accurately, *de nihilo*." This is amusing. Nothing can arise *de nihilo—ex nihilo nihil fit*. Dr. Willoughby will admit that this is good science. It is clear that though he is anxious to affirm that specific diseases can only arise in one way, he is still haunted by a harassing doubt. The old question which has puzzled children, as well as doctors, rings in Dr. Willoughby's innermost ears, although there may be no sound of it in the external medical atmosphere of the present day. "How did the first case of every specific disease arise?" whispers the still unlaid ghost of medical scepticism. "How?" echoes Dr. Willoughby's honest but unsatisfied consciousness. The cause of specific diseases is, says Dr. Willoughby, "evidently something material and external to the body entering the organism by the alimentary, respiratory, or other mucous and absorbent surface, or by some breach in the cutaneous covering." And again, "with the exception of one or two small groups, they are all more or less demonstratively or presumably communicated from one individual to another." Now why does Dr. Willoughby use such words as "presumably," "evidently," and so on, if it is quite certain that what he calls specific diseases never arise *de novo*, but always result from the transmission of specific microbes from one individual to another? The truth is that Dr. Willoughby, in common with every other honest scientist, is not absolutely certain about every point in the natural history of so-called specific diseases. He gives us the current hypotheses; and if human industry and care may be relied upon, those hypotheses, at least point in the direction of scientific truth.

